# Phenotypic variation in sexually and asexually recruited individuals of the Baltic Sea endemic macroalga *Fucus radicans*: in the field and after growth in a common-garden

**DOI:** 10.1186/1472-6785-12-2

**Published:** 2012-02-22

**Authors:** Kerstin Johannesson, Helena Forslund, Nastassja Åstrand Capetillo, Lena Kautsky, Daniel Johansson, Ricardo T Pereyra, Sonja Råberg

**Affiliations:** 1 Department of Marine Ecology - Tjärnö, University of Gothenburg, Strömstad, Sweden; 2 Department of Botany, Stockholm University, Stockholm, Sweden

**Keywords:** Phenotypic traits, Inherited variation, Foundation species, Ecosystem function

## Abstract

**Background:**

Most species of brown macroalgae recruit exclusively sexually. However, *Fucus radicans*, a dominant species in the northern Baltic Sea, recruits new attached thalli both sexually and asexually. The level of asexual recruitment varies among populations from complete sexual recruitment to almost (> 90%) monoclonal populations. If phenotypic traits have substantial inherited variation, low levels of sexual activity will decrease population variation in these traits, which may affect function and resilience of the species. We assessed the level of inherited variation in nine phenotypic traits by comparing variation within and among three monoclonal groups and one group of unique multilocus genotypes (MLGs) sampled in the wild.

**Results:**

Of the nine phenotypic traits, recovery after freezing, recovery after desiccation, and phlorotannin content showed substantial inherited variation, that is, phenotypic variation in these traits were to a large extend genetically determined. In contrast, variation in six other phenotypic traits (growth rate, palatability to isopod grazers, thallus width, distance between dichotomies, water content after desiccation and photochemical yield under ambient conditions) did not show significant signals of genetic variation at the power of analyses used in the study. Averaged over all nine traits, phenotypic variation within monoclonal groups was only 68% of the variation within the group of different MLGs showing that genotype diversity does affect the overall level of phenotypic variation in this species.

**Conclusions:**

Our result indicates that, in general, phenotypic diversity in populations of *Fucus radicans *increases with increased multilocus genotype (MLG) diversity, but effects are specific for individual traits. In the light of *Fucus radicans *being a foundation species of the northern Baltic Sea, we propose that increased MLG diversity (leading to increased trait variation) will promote ecosystem function and resilience in areas where *F. radicans *is common, but this suggestion needs experimental support.

## Background

In most ecosystems, increased species richness tends to support ecosystem function [1-3], but in ecosystems dominated by one or a few foundation species - such as seagrass meadows, seaweed belts, and stands of forest trees - species diversity may be less important while instead phenotypic diversity within the foundation species is likely to affect properties of the ecosystem as a whole, such as function and resilience [4]. For example, a population of a habitat-forming species in which the individuals are phenotypically different will create a more complex habitat than one in which phenotypes are all alike, and most likely the more complex habitat will attract more associated species [5]. Many foundation species both in terrestrial and aquatic habitats form new individuals by either sexual or asexual reproduction, and populations formed by one or a few different clones will provide less phenotypic variation than multi-clonal populations, whenever phenotypic traits are largely explained by genetic variation.

Experimental studies with plant species of mixed sexual and asexual recruitment have shown that, for example, primary production, carbon storage, nutritional load of surrounding soils and diversity of associated species increase whenever genotype diversity of populations increases [6]. Indeed, effects are sometimes comparable in magnitude to effects of increased species diversity [7]. As for species biodiversity, the link between genotype diversity and ecosystem function is suggested to be either a sampling effect, or a complementarity effect, or a combination of both. A sampling effect is the consequence of adding more genotypes, and thereby increase the likelihood that more profitable traits are included in the population [8,9]. However, increased genotype diversity may also improve performance of a population through complementarities among genotypes, that is, individuals of different genotypes facilitate performance of each other [10,11].

In marine ecosystems, studies that manipulate MLG diversity in experimental plots have been performed in seagrass (*Zostera marina*) meadows. Such experiments have shown that meadows of high MLG diversity (many different clones) better resist periods of intense grazing, grazing from specific grazers, extreme weather conditions, and have higher productivity and biomass of associated invertebrate fauna than meadows of low MLG diversity (few clones) [4,12,13]. The positive correlation between MLG diversity and ecosystem performance in *Zostera marina *have been attributed to both sampling and complementarity effects [4,13].

*Fucus *species are important foundation species of coastal waters, providing shelter and food for various invertebrate and fish species [14-16]. In the northern parts of the semi-enclosed Baltic Sea, two species of *Fucus *are present, *F. vesiculosus *L. and *F. radicans *Bergström & Kautsky [17,18] and here, in addition to sexual reproduction, individuals of both species have the capacity to form new attached thalli from adventitious branches that come loose from the thallus, are swept away by water motions, and later re-attach to the substratum by formation of rhizoids [19]. Asexual recruitment of attached thalli is hitherto not found outside the Baltic Sea, and the reason for this is not clear. A possible reason why it exists inside the Baltic Sea may be that zygote formation is partly impeded by the low ambient salinity in the northern part of the Baltic (< 4-5‰) [20,21]. However, asexual reproduction is overall much more common in *F. radicans *than in *F. vesiculosus *also in this area. In addition, populations of *F. radicans *living in similar salinities may differ a lot in the prevalence of asexual recruitment, ranging from populations strongly dominated by one clone to populations with only sexually recruited thalli [22]. A female clone, in particular, is exceptionally widespread and dominates many populations over much of the distribution of *F. radicans*.

The aim of the present study was to find out if phenotypic variation among thalli of *F. radicans *are to any substantial extent explained by genetic variation, or if most of observed trait variation in nature is non-genetic variation induced by variable environmental conditions. If there is substantial inherited phenotypic variation, we would expect that populations of low MLG diversity (few clones), for example, the nearly monoclonal populations found in some areas, will have lower capacity with respect to ecosystem function and resilience than populations of high MLG diversity (many clones present in the same population), as has been shown in *Zostera marina *and terrestrial plant species [6,13].

In this study we used thalli of *F. radicans *sampled in two areas about 30 km apart in the northern part of the Bothnian Sea (northern Baltic Sea). The multilocus genotype of each thalli was determined by microsatellite analysis and thalli were thereafter arranged into four groups; three monoclonal groups made up of the three most common MLGs in the samples, and one group composed of singletons, that is, unique MLGs found in single copies and presumably of sexual origin. We measured phenotypic variation within and among groups in nine phenotypic traits and found phenotypic variation in three of them showing evidence of strong inherited variation.

## Results and discussion

Using 5 microsatellite loci we genotyped 147 thalli of *F. radicans *that were picked from separate holdfasts widely distributed over two sampling areas (approx. 200 m^2 ^each and 30 km apart) in the northernmost part of the species' distribution. Among these thalli we found three clones that were represented by 14-93 thalli each and we selected 9 equally sized thalli per clone to make up our three monoclonal groups. In addition, we found 12 individuals of unique genotypes and selected 9 of these to represent a group of unique MLGs. In all individuals of all four groups we analysed phenotypic variation in thallus width and distance between dichotomies ("branches"), the two most important morphological traits used to separate *F. radicans *and *F. vesiculosus *in the field [17]. We furthermore measured phlorotannin concentrations and palatability to grazing from isopods, traits suggested to be important to local adaptation and to the geographic distribution of *F. radicans *[23]. These four traits were measured a few days after sampling, or for grazing, a few months later (late August), but on tissue grown before sampling, and hence likely included phenotypic variation that was already present in the field.

The total phenotypic variation we found in the traits measured without a common garden treatment were composed of two different parts (*i*) variation among thalli of the same MLG, and (*ii*) variation among MLGs. Variation among thalli and within MLGs must be environmentally induced and caused by thalli being grown in different microhabitats. Under the assumption that thalli of the same MLG were randomly distributed over the sampling area, differences among MLGs indicated inherited variation. The assumption of random distribution of individuals of the same MLG is supported by our observation that all the three common clones were present in both sampling areas, and that we in a detailed study of another site (of similarly size as the two sampled sites of the current study) found separate clones of *F. radicans *to be randomly distributed in space (Pereyra et al. pers. communication). For the traits measured at this stage, we found highly significant differences among MLGs in phlorotannin content while differences were non-significant in palatability to grazers, thallus width and distance between dichotomies (Table 1). This strongly suggests that variation in phlorotannin content is, to a large part, inherited.

**Table 1:** ANOVA statistics (*P*-values) indicating differentiation of traits.

	*No common garden treatment*				*Common garden treatment*				
Differentiation	Thallus width	Distance between dichotomies	Phlorotannin content	Palatability to grazers	Photochemical yield	Photochemical yield after desiccation	Photochemical yield after freezing	Water content after desiccation	Growth during 3 months
ANOVA clones	0.55	0.1	**0.0001**	0.49	0.08	0.059	**0.002**	0.99	0.36
Post-hoc (SNK)									
Clone-0 vs Clone-1			*P *< 0.05				*P *< 0.05		
Clone-0 vs Clone 4						0.05 <*P *< 0.10	*P *< 0.05		
Clone-1 vs Clone 4			*P *< 0.05			0.05 <*P *< 0.10			
Effect			Clone-1 higher			Clone-4 higher	Clone-0 lower		

Variation of phenotypic trait values for nine different traits are compared among the 3 monoclonal groups and significant results further evaluated with SNK posthoc test. Trait values were used untransformed with low deviations from homogeneous variances. Significant values are indicated in bold

The initial measurements were followed by all thalli being cultivated in a common garden environment (large outdoor seawater tanks) starting in late May 2009. In September four additional phenotypic traits were measured; growth during 90 days, photochemical yield under normal conditions, and photochemical yield and water content after desiccation, and in January 2010 photochemical yield after freezing. These traits have physiological relevance and are likely to affect fitness of thalli under different environmental conditions. For these traits, all the measurements were performed on new tissue formed during the common garden treatment. As macroalgae do not possess a vascular system that connects different parts of the thallus, the metabolic status of old tissue is not easily transferred to new tissue [24]. Hence, it is most likely that for these traits environmental variation induced in the field was largely eliminated in the new grown tissue used in the measurements. Thus, in this case, differences among MLGs indicated genetic differences also if the assumption of random distribution of thallus of the same MLGs in the field would not hold. In this comparison we found a highly significant difference among MLGs in the photochemical yield after freezing, and MLGs were also nearly significantly different in photochemical yield after desiccation (Table 1). These differences show that variation in recovery from freezing stress, and desiccation stress, has a genetic component.

**Figure 1:**
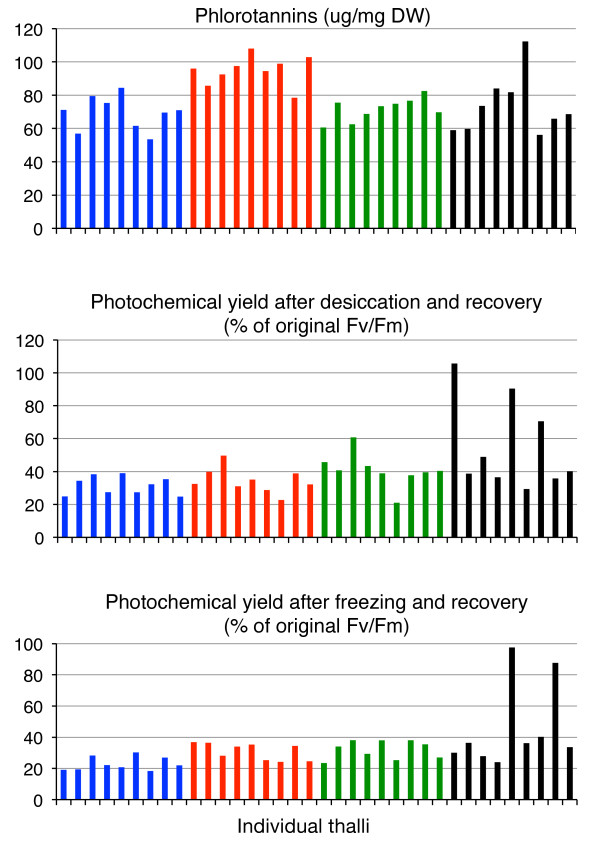
**Trait values for traits that showed significant inherited variation**. Values for each individual thalli are indicated (separate bars) and groups are blue: clone-0, red: clone-1, green: clone 4 and black unique multilocus genotypes.

We also examined the variation within the group of unique MLGs and found these to be significantly larger than within the monoclonal groups for the same three phenotypic traits, and this observation strongly support the conclusion of inherited variation in resistance to desiccation, resistance to freezing and content of phlorotannins (Table 2, Figure 1). Furthermore, none of these traits were significantly correlated with each other, which tentatively suggest that they are inherited as fully independent traits (Table 3). A notable result was that only a few of the individuals contributed to the increased phenotypic variation in the group of unique MLGs (Figure 1). In addition, the MLGs that contributed the most to the deviation where different for the different traits with the exception of one MLG that were both highly resistant to freezing and to desiccation (Figure 1). The reason for the rather dramatic differences in trait values of individual MLGs is unclear but may perhaps be explained by relatively few quantitative trait loci (QTLs) or genomic regions being involved in these specific traits. While the results for the remaining traits did not indicate strong inherited variation, it is important to underline that sample sizes were too small to identify modest or low levels of inherited phenotypic variation.

**Table 2:** Cochran's test of homogeneous variances among experimental groups.

	*No common garden treatment*				*Common garden treatment*				
Population	Thallus width	Distance between dichotomies	Phlorotannin content	Palatability to grazers	Photochemical yield	Photochemical yield after desiccation	Photochemical yield after freezing	Water content after desiccation	Growth after 3 months
Clone-0	0.40	24.33	104.98	0.01	21.30	31.18	19.02	1.41	7.98
Clone-1	0.16	4.89	77.44	0.01	16.89	59.06	28.89	9.56	11.24
Clone-4	0.38	22.52	55.06	0.01	21.45	119.31	35.24	19.64	6.21
Uniques	0.58	15.62	308.41	0.00	29.68	743.91	728.38	27.68	8.80
Cochran's C	0.38	0.36	0.56*	0.32	0.33	0.78*	0.90*	0.47	0.33

*Significant at *P *< 0.05, critical C = 0.52. Phenotypic trait variances are indicated for three monoclonal groups and one group of unique multilocus genotypes and compared with Cochran's C. Significant heterogeneity in variances are indicated with a C value in bold

**Table 3:** Correlation among traits.

	*N*	Correlation coefficient	*P*
Growth rate vs Phlorotannin content	11	-0.53	0.05-0.1
Photochemical yield after freezing vs Thallus width	12	0.51	0.05-0.1
Photochemical yield after desiccation vs Water content after 2 h of desiccation	12	0.54	0.05-0.1

Correlation coefficients are shown for those phenotypic traits that showed marginally significant correlations (0.05 <*P *< 0.1). (No correlations were significant at α = 0.05). All thalli from the group of unique multilocus genotypes were included in the analyses together with one randomly chosen thallus of each of the three monoclonal groups. *P*-values are not corrected for multiple testing

Studies in the sister species *F. vesiculosus *have shown that the inherited part of the variation in growth rate is low while it is high in phlorotannin content [25,26], and this is similar to what we found for *F. radicans*. Phlorotannins provide a defence towards grazing, in particular by isopods, in *F. vesiculosus *[25-27], and this seems most likely the case also in *F. radicans*. We found only a weak and non-significant negative correlation (r = -0.02) between phlorotannin content and palatability to isopod grazers in the present study, but we did find an almost significant negative correlation with growth rate (Table 3), suggesting phlorotannin production to be costly.

Isopod abundance is low in the Bothnian Sea compared to in the Baltic Proper [28], and grazing pressure from isopods seems mostly low or absent in most areas where *F. radicans *is present [18]. In addition, phlorotannin production is known to be costly [29], this study], and, thus there is probably a trade-off favouring a low phlorotannin production in areas of low grazing pressure. Indeed, in choice experiments, isopods graze substantially more on *F. radicans *than on *F. vesiculosus *[18], which suggests that phlorotannin content is generally lower in *F. radicans *than in *F. vesiculosus*. As we now show, there is genetic variation present in this trait in *F. radicans *and hence selection may favour genotypes of high or low phlorotannin content depending on the local grazing pressure. A less likely alternative (owing to costs of production) is that the high phlorotannin production we found in some of the clones is a remnant from the more saline Littorina Sea period of the Baltic Sea a few thousand years ago, when isopods were likely more common in the Bothnian Sea than they are today. It has also been suggested that phlorotannins protect the thalli from UV radiation [30] but this seems of less contemporary importance as *F. radicans *in the Baltic lives permanently submerged.

Perhaps still more intriguing is the finding that populations of *F. radicans *contain MLGs that fully recover from freezing dowo-15°C or desiccation during 3 h, while this species lives submerged and only very occasionally may be exposed to desiccation or freezing during periods of extreme low water in the Bothnian Bay. Indeed, it seems unlikely that selection favouring such characters would be more than very occasional. However, the ancestral population of *F. radicans*, that is, the population of *F. vesiculosus *that entered the Baltic Sea about 6000 years ago, and from which *F. radicans *evolved [31], came from intertidal habitats in the North Sea. Here, resistance to freezing and desiccation is critical to survival as most individuals live emerged during periods of low water. In fact, populations of *F. vesiculosus *from the North Sea show much higher tolerance to desiccation than Baltic populations of this species [32], and these differences seem largely inherited [33]. The high tolerance of single MLGs of *F. radicans *to desiccation and freezing may be what is left of a very common genotype in the ancestral gene pool entering the Baltic Sea. That this genotype is still present in low frequencies may be due to local and temporal positive selection during rare events of extreme low water level. Alternatively or in addition, there may be epistatic or pleiotrophic effects in combination with weak negative selection, or simply that genetic drift only very slowly replace near neutral variation in large populations.

## Conclusions

In the experimental populations of this study, phenotypic variation in monoclonal stands were significantly lower than in the multi-clonal group for three of nine phenotypic traits (Table 2), and, as expected, these three traits were the ones that showed inherited phenotypic variation (Table 1). In the seagrass, *Zostera marina*, and in terrestrial plant species, reduction in trait variation in stands of low clonal diversity impedes ecosystem functions such as resilience to extreme stress, productivity, biomass of associated biodiversity, and resistance to grazing [4,34,35]. Such a scenario seems likely also in *F. radicans*, that is, when trait variation is reduced, in stands of low clonal diversity, ecosystem function will decrease. In nature, we have not yet found any completely monoclonal population of *F. radicans*, but several populations have a composition with one clone (clone-4 of this study) constituting 70-90% of the thalli [22]. Consequently, these populations are likely to show decreased phenotypic variation in any trait with substantial inherited variation. If, more specifically, the loss of variation in the traits that showed inherited variation in the present study (phlorotannin content, resistance to freezing, and resistance to desiccation) affect ecosystem function or resilience of nearly monoclonal populations of *F. radicans *in their current situation, remains an open question. However, due to further reduces salinities in the northern Baltic Sea under a warmer climate [36,37] the long term survival and sustainability of *F. radicans *may be challenged and redistribution of the species into the southern and western areas of the Baltic Sea a necessity. If so, grazing pressures and tidal conditions will change and inherited variation promoting tolerance to grazing, freezing and desiccation, may be highly favourable for long-term species survival.

## Methods

### Sampling, cultivation and acclimatization

Sampling was made at two sites; Hällkalla (63°25'N, 20°57'E) and Södra Vallgrund (63°09'N, 21°13'E) 30 km apart, in May 2009 using Scuba-diving. The individuals were collected at 2-6 m depth and from 200 m^2 ^of area in both sites. These sites represent the northernmost distribution of the species and salinity is very low (3-4 practical salinity units, psu). The thalli were transported alive to the Askö Laboratory (58°49'N, 17°38'E), on the Swedish coast of the northern Baltic Proper. Tissue for analysis of phlorotannin content and genotype were sampled immediately upon arrival. All 147 thalli were thereafter genotyped using microsatellites (see below) and 9 individuals of each of three clones, and in addition 9 individuals of unique genotypes, in total 36 thalli, were chosen for further analyses of traits. Whole thalli were stored in outdoor tanks at ambient temperature and light conditions (a common garden). The salinity in the tanks was 6.5 psu, that is, somewhat higher than at the sampling sites, but earlier observations had shown that *F. radicans *thalli perform well in this salinity (H. Forslund pers. obs). Thallus width, distances between dichotomies and resistance to grazing were assessed for the 36 chosen thalli soon upon arrival to Askö. Following cultivation of the thalli in the tanks over the summer we measured growth rate, normal photochemical yield and resistance to desiccation on new-grown tissue in September 2009. In December the thalli were transferred to indoor tanks at 5°C, 6.5 psu and a photon-flux density (PFD) of 50 μmol photons m^-2 ^s^-1^, and 16:8 h light:dark photoperiod. In January 2010 a freezing experiment was performed measuring photochemical yield in new-grown tissue after exposure to freezing.

### Genetic analysis

We genotyped five microsatellite loci in all 147 thalli. Silica gel dried tissue from each thalli was pulverized in a milling instrument (Mixer Mill MM 301, Retsch) for 30 s at a frequency of 25 s^-1^. DNA was extracted using Viogene's Plant Genomic DNA Extraction Miniprep System (Viogene, Sunnyvale, CA, USA) according to the manufacturer's protocol. (For details on PCR protocols see [22]). The amplified fragments were separated in a capillary automated sequencer (CEQ8000) and sized using CEQ software. Samples were genotyped at the loci L20, L38, L58, L85 and L94 [38]. These 5 loci have earlier showed powerful enough to discriminate between different clones [19,22,31]. The three clones most common in the samples were heterozygote in zero (clone-0), one (clone-1), and four (clone-4) out of 5 microsatellite loci. Clone-4 (a female clone) has been identified in earlier studies and has an extensive distribution along the northern Swedish and Finnish coasts. Clone-0 (a male clone) was recently found in one additional site at the Swedish coast (Skagsudde, D. Johansson, pers. obs.), while clone-1 (also a male clone) is very common in the sampling area of the present study but has yet not been found elsewhere.

### Phenotypic traits

#### Thallus width and distance between dichotomies

Thallus width was measured on three branches of each individual between the first and the second dichotomy. Distance between dichotomies was measured from the second oldest to the third oldest dichotomy and so forth up to the seventh dichotomy on a branch [17].

#### Phlorotannin content

Pieces of algal tissue were freeze-dried, homogenized and 12-15 mg powder was extracted in 1.5 ml aqueous acetone (60%) on a vortex during 1 h. Following centrifugation 100 μl of the supernatant was diluted with MQ water to a volume of 8 ml and 0.5 ml Folin-Ciocalteu's reagent (Merck, Art. 109001) and 1.5 ml sodium carbonate was added to the sample. Total phlorotannin level was measured as absorbance at 740 nm after 2 h of incubation in the dark using a spectrophotometer and phloroglucinol (1,3,5-Trihydroxybenzene, Sigma, Art. 6099-90-7) as a standard [39].

#### Palatability to grazers

Two pieces of each of the 36 thalli (0.4-0.6 g wet weight) were put into separate compartments in a small flow-through container, hanging from a jetty for four days at ambient temperature (15-16°C) and salinity (6.0-6.1 psu). Four isopod grazers (*Idotea baltica*) collected at Askö were added to one of the compartments. We estimated the amount of grazed tissue of each thalli from the loss in wet weight of the grazed part plus the gain of tissue in the control (due to growth during the experiment) divided by the dry weight of all four grazers in one container.

#### Maximum photochemical yield under normal conditions

Tissue formed during a common garden period of 4 month were sampled from all thalli, emerged at 15°C and protected from desiccation by being placed in fully hydrated plastic boxes on damp paper towels. Maximum photochemical yield (Fv/Fm - variable fluorescence/maximum fluorescence) was measured in dark-adapted tissue using a pulse amplitude-modulated fluorometer (PAM; Watlz, Effeltrich, Germany). The tissue was adapted to darkness during 10 min of complete darkness before and during measurements.

#### Water content after desiccation

We removed apical vegetative pieces (150 mg wet weight) from each thalli and left these to desiccate on a mesh screen for 2.5 h at 15°C in light. The mesh was used to reduce variation in drying rate caused by accumulation of water between the tissue and the surface. The weight was recorded every 15 min. Variation among thalli in water loss were largest after 2 h of desiccation and we used these values as a proxy for this trait.

#### Maximum photochemical yield after desiccation

When desiccation had ceased after 2.5 h, the apical pieces were transferred to seawater (6.5 psu) and left to recover for 2 h under light conditions. Prior to desiccation and at the end of the recovery period maximum photochemical yield was measured with a PAM (same procedure as indicated above). Maximum photochemical yield after recovery were expressed as percentage of yield prior to desiccation.

#### Maximum photochemical yield after freezing

The experimental conditions for measuring recovery from freezing were similar to the measurements of maximum photochemical yield after desiccation. The only differences were that the mesh screens were maintained at -15°C. In addition, periods of exposure as well as recovery were set to 3 h. Notably, there was no freeze-drying tendencies during the period of low temperature and the samples were wet and soft once they were warmed up after freezing. Maximum photochemical yield after freezing and recovery were expressed as percentage of the yield prior to the stress.

#### Growth rate

Thalli were marked with a thin thread 10 mm from the apex and growth rate was calculated from measuring the distance between the thread and the apex after 90 days of growth in the common garden system.

## Authors' contributions

SR, HF, NÅC, LK, DJ, RTP and KJ designed research. RTP and DJ performed field sampling and microsatellite analyses. SR, HF, NÅC and LK conducted ecological experiments. DJ and KJ performed molecular and statistical analysis, respectively. KJ drafted the manuscript. All authors improved and finally approved the manuscript.

## Authors' information

SR, HF and NÅC work as postdoc, PhD student and master student, respectively in the research group led by LK on *Fucus *ecology. DJ and RTP work as PhD student and researcher, respectively, in the group led by KJ focusing on population genetics of *Fucus*. Both groups are part of the BaltGene project under the BONUS programme, and KJ's group is also partner in the Linnaeus Centre for Marine Evolutionary Biology (CeMEB). BaltGene and CeMEB are coordinated by KJ.

## References

[B1] Hooper DU Chapin FS Ewel JJ Effects of biodiversity on ecosystem functioning: a consensus of current knowledge Ecol Monographs 2005 75 3 35 10.1890/04-0922

[B2] Balvanera P Pfisterer AB Buchmann N He J-S Nakashizuka T Raffaelli D Schmid B Quantifying the evidence for biodiversity effects on ecosystem functioning and services Ecol Lett 2006 9 1146 1156 10.1111/j.1461-0248.2006.00963.x 16972878

[B3] Cardinale BJ Wright JP Cadotte MW Impacts of plant diversity on biomass production increase through time because of species complementarity Proc Natl Acad Sci USA 2007 104 18123 18128 10.1073/pnas.0709069104 17991772 PMC2084307

[B4] Reusch TBH Ehlers A Hämmerli A Worm B Ecosystem recovery after climatic extremes enhanced by genotypic diversity Proc Natl Acad Sci USA 2005 102 2826 2831 10.1073/pnas.0500008102 15710890 PMC549506

[B5] Johnson MTJ Agrawal AA Plant genotype and environment interact to shape a diverse arthropod community on evening primrose (*Oenothera biennis*) Ecology 2005 86 874 885 10.1890/04-1068

[B6] Bailey JK From genes to ecosystems: a genetic basis to ecosystem services Popul Ecol 2011 53 47 52 10.1007/s10144-010-0251-4

[B7] Crutsinger GM Collins MD Fordyce JA Gompert Z Nice CC Sanders NJ Plant genotypic diversity predicts community structure and governs an ecosystem process Science 2006 313 966 968 10.1126/science.1128326 16917062

[B8] Huston MA Hidden treatments in ecological experiments: re-evaluating the ecosystem function of biodiversity Oecologia 1997 110 449 460 10.1007/s004420050180 28307235

[B9] Tilman D Lehman CL Thomson KT Plant diversity and ecosystem productivity: theoretical considerations Proc Natl Acad Sci USA 1997 94 1857 1861 10.1073/pnas.94.5.1857 11038606 PMC20007

[B10] Loreau M Hector A Partitioning selection and complementarity in biodiversity experiments Nature 2001 412 72 76 10.1038/35083573 11452308

[B11] Norberg J Swaney DP Dushoff J Lin Jr Levin SA Phenotypic diversity and ecosystem functioning in changing environments: a theoretical framework Proc Natl Acad Sci USA 2001 98 11376 11381 10.1073/pnas.171315998 11535803 PMC58737

[B12] Hughes AR Stachowicz JJ Genetic diversity enhances the resistance of a seagrass ecosystem to disturbance Proc Natl Acad Sci USA 2004 101 8998 9002 10.1073/pnas.0402642101 15184681 PMC428461

[B13] Hughes AR Best RJ Stachowicz JJ Genotypic diversity and grazer identity interactively influence seagrass and grazer biomass Mar Ecol Prog Ser 2010 403 43 51

[B14] Råberg S Kautsky L A comparative biodiversity study of the associated fauna of perennial fucoids and filamentous algae Estuar Cost Shelf Sci 2007 73 249 58 10.1016/j.ecss.2007.01.005

[B15] Wikström SA Kautsky L Structure and diversity of invertebrate communities in the presence and absence of canopy-forming *Fucus vesiculosus *in the Baltic Sea Estuar Cost Shelf Sci 2007 72 168 76 10.1016/j.ecss.2006.10.009

[B16] Kautsky H Kautsky L Kautsky N Kautsky U Lindblad C Aspects on *Fucus vesiculosus *in the Baltic Sea Acta Phytogeo Suec 1992 78 33 48

[B17] Bergström L Tatarenkov A Johannesson K Jönsson R Kautsky L Genetic and morphological identification of *Fucus radicans *sp. nov. (Fucales, Phaeophyceae) in the brackish Baltic Sea J Phycol 2005 41 1025 1038 10.1111/j.1529-8817.2005.00125.x

[B18] Forslund H Eriksson O Kautsky L Grazing and geographic range of the Baltic seaweed Fucus radicans (Phaeophyceae) Marine Biology Research 8 386 394

[B19] Tatarenkov A Bergström L Jönsson RB Serrão EA Kautsky L Johannesson K Intriguing asexual life in marginal populations of the brown seaweed *Fucus vesiculosus* Mol Ecol 2005 14 647 51 10.1111/j.1365-294X.2005.02425.x 15660953

[B20] Serrão EA Brawley SH Hedman J Kautsky L Samuelsson G Reproductive success of *Fucus vesiculosus *(Phaeophyceae) in the Baltic Sea J Phycol 1999 35 254 69 10.1046/j.1529-8817.1999.3520254.x

[B21] Brawley SH The fast block against polyspermy in foucoid algae is an electrical block Dev Biol 1991 144 94 106 10.1016/0012-1606(91)90482-I 1995405

[B22] Johannesson K Johansson D Larsson KH Huenchuñir Perus J Forslund AH Kautsky L Pereyra RT Frequent clonality in fucoids (*Fucus radicans *and *F. vesiculosus*; fucales, phaeophyceae) in the Baltic Sea J Phycol 2011 47 990 998 10.1111/j.1529-8817.2011.01032.x 27020180

[B23] Wahl M Jormalainen V Eriksson BK Coyer JA Molis M Schubert H Dethier M Karez R Kruse I Lenz M Pearson G Rodhe S Wikström S Olsen JL Stress ecology in *Fucus*: abiotic, biotic and genetic interactions Adv Mar Biol 2011 59 37 105 21724018 10.1016/B978-0-12-385536-7.00002-9

[B24] Toth GB Pavia H Water-borne cues induce chemical defense in a marine alga (*Ascophyllum nodosum*) Proc Natl Acad Sci USA 2000 97 14418 14420 10.1073/pnas.250226997 11106371 PMC18933

[B25] Jormalainen V Ramsay T Resistance of the brown alga Fucus vesiculosus to herbivory Oikos 2009 118 713 722 10.1111/j.1600-0706.2008.17178.x

[B26] Jormalainen V Honkanen T Variation in natural selection for growth and phlorotannins in the brown alga *Fucus vesiculosus* J Evol Biol 2004 17 807 820 10.1111/j.1420-9101.2004.00715.x 15271080

[B27] Haavisto F Valikangas T Jormalainen V Induced resistance in a brown alga: phlorotannins, genotypic variation and fitness costs for the crustacean herbivore Oecologia 2010 162 685 695 10.1007/s00442-009-1494-7 19921521

[B28] Nilsson J Engkvist R Persson LE Long-term decline and recent recovery of *Fucus *populations along the rocky shores of southeast Sweden, Baltic Sea Aqua Ecol 2004 38 587 598 10.1007/s10452-004-5665-7

[B29] Pavia H Toth G Åberg P Trade-offs between phlorotannin production and annual growth in natural populations of the brown seaweed *Ascophyllum nodosum* J Ecol 1999 87 761 771 10.1046/j.1365-2745.1999.00397.x

[B30] Pavia H Cervin G Lindgren A Åberg P Effects of UV-B radiation and simulated herbivory on phlorotannins in the brown alga *Ascophyllum nodosum* Mar Ecol Prog Ser 1997 143 139 146

[B31] Pereyra RT Bergström L Kautsky L Johannesson K Rapid speciation in a newly opened postglacial marine environment, the Baltic Sea BMC Evol Biol 2009 9 70 10.1186/1471-2148-9-70 19335884 PMC2674422

[B32] Pearson GA Kautsky L Serrão EA Recent evolution in Baltic *Fucus vesiculosus*: reduced tolerance to emersion stresses compared to intertidal (North Sea) populations Mar Ecol Prog Ser 2000 202 67 79

[B33] Pearson GA Hoarau G Lago-Leston A Coyer JA Kube M Reinhardt R Henckel K Serrão ETA Corre E Olsen JL An expressed sequence Tag analysis of the intertidal brown seaweeds *Fucus serratus *(L.) and *F. vesiculosus *(L.) (Heterokontophyta, Phaeophyceae) in response to abiotic stressors Mar Biotechnol 2010 12 195 213 10.1007/s10126-009-9208-z 19609612

[B34] Hughes AR Stachowicz JJ Ecological impacts of genotypic diversity in the clonal seagrass *Zostera marina* Ecology 2009 90 1412 1419 10.1890/07-2030.1 19537560

[B35] Ehlers A Worm B Reusch TBH Importance of genetic diversity in eelgrass *Zostera marina *for its resilience to global warming Mar Ecol Prog Ser 2008 355 1 7

[B36] Meier HEM Baltic Sea climate in the late twenty-first century: a dynamical downscaling approach using two global models and two emission scenarios Clim Dyn 2006 27 39 68 10.1007/s00382-006-0124-x

[B37] Meier H Eilola K Almroth E Climate-related changes in marine ecosystems simulated with a 3-dimensional coupled physical-biogeochemical model of the Baltic Sea Clim Res 48 31 55

[B38] Engel CR Brawley S Edwards KJ Serrão E Isolation and cross-species amplification of microsatellite loci from the fucoid seaweeds *Fucus vesiculosus, F. serratus*, and *Ascophyllum nodosum *(Heterokontophyta, Fucaceae) Mol Ecol Notes 2003 3 180 182 10.1046/j.1471-8286.2003.00390.x

[B39] van Alstyne KL Comparison of three methods for quantifying brown algal polyphenolic compounds J Chem Ecol 1995 21 45 58 10.1007/BF02033661 24233566

